# Using iVR to deliver optimal psychotherapy experience—current perspectives on VRET for acrophobia

**DOI:** 10.3389/fpsyg.2024.1491622

**Published:** 2024-12-05

**Authors:** Kristína Kvapil Varšová, Vojtěch Juřík

**Affiliations:** ^1^Department of Psychology, Faculty of Arts, Masaryk University, Brno, Czechia; ^2^Institute of Computer Aided Engineering and Computer Science, Faculty of Civil Engineering, Brno University of Technology, Brno, Czechia

**Keywords:** iVR, virtual reality, VRET, exposure, CBT, acrophobia, CIVE

## Abstract

Immersive Virtual Reality (iVR) presents a promising avenue for treating acrophobia through Virtual Reality Exposure Therapy (VRET). This paper explores the current state of VRET for acrophobia, identifying significant technological and practical barriers that limit its effectiveness and hinder widespread adoption. Key challenges include the need for more advanced and realistic user experiences, and for the integration of biofeedback mechanisms. Additionally, the role of therapists remains crucial, as therapist-led VRET sessions demonstrate better outcomes compared to automated interventions. The potential of Collaborative Immersive Virtual Environments (CIVEs) to enhance VRET by simulating real-life interactions and improving patient-therapist engagement is also discussed. Future research should focus on developing comprehensive guidelines for CIVE design and evaluating integrated VRET and CIVE systems for treating phobias, including acrophobia. Addressing these issues will enhance the therapeutic experience, making VRET a more effective and accessible tool for treating acrophobia.

## 1 Introduction

iVR is an advanced technology known for its three-dimensional, multisensory, and interactive experiences, replacing physical environments and fostering a strong sense of presence (Slater, 2018). iVR presents significant potential within the mental health field as it provides a unique method of immersing individuals in simulated difficult scenarios (Emmelkamp and Meyerbröker, 2021). Particularly, the potential of iVR in treating anxiety and stress-related disorders has been recognized by the field of psychology. iVR has been adopted as a promising method for managing anxiety and phobias (Schröder et al., 2023; Wray et al., 2023), including acrophobia (Roesmann et al., 2023; Wechsler et al., 2019). Acrophobia is defined by severe anxiety when one is near heights, aversion to heights, and consequent impairment of functioning. When in a high area, people with this condition may experience panic episodes and become too anxious to safely descend. Depending on how severe it is, this condition may lead to a restricted lifestyle or impede some types of work (Huppert et al., 2020).

Numerous significant technological interventions in this field incorporate principles of Cognitive Behavioral Therapy (CBT) through diverse digital media (Fairburn and Patel, 2017). CBT, mainly exposure therapy, has become a popular technique for treating acrophobia (Arroll et al., 2017; Chard and van Zalk, 2022; Chou et al., 2021). With the use of visual, aural, and sensory stimuli, VRET mimics *in vivo* exposure as a potential therapy option. Additionally, augmented reality (AR) has been applied in the treatment of phobic disorders, showing promising results (Hasan et al., 2023). When compared to traditional (*in vivo*) exposure, VRET or AR offer practitioners an advantage because it is nearly impossible to fine-tune exposure levels in real-world circumstances (Chou et al., 2021; Dellazizzo et al., 2020; Palau-Batet et al., 2024). A unique, controlled exposure made possible by iVR technology offers the chance to face fear in a secure environment and produce clinically meaningful effects, with data demonstrating notable improvements in treatment outcomes (Donker and Heinrichs, 2023; Varšová et al., 2024; Wechsler et al., 2019). In common procedures for VRET treatment of acrophobia, individuals undergo immersive VR programs with or without (automated treatment) a therapist present. A virtual or real coach guides them through graded, height-related activities designed to help confront and reduce their phobia. The participant’s virtual body is calibrated using data from head and hand VR trackers, allowing them to stand and walk within the virtual environment (Freeman et al., 2018; Lindner et al., 2017; Varšová et al., 2024; Yang and Wang, 2022).

Despite numerous advances in iVR and VRET, several barriers persist in achieving an optimal experience and realizing the full potential of iVR technology in VRET for acrophobia. This paper aims to identify these barriers and discuss the current state of technological and process advancements. Most importantly, we seek to highlight potential future directions for VRET and explore ways to enhance its effectiveness.

## 2 Barriers to the use of VRET in practice for the treatment of acrophobia

iVR-assisted psychotherapy offers significant promise as a pioneering example of how digital tools can enhance mental health care, particularly through VRET (Emmelkamp and Meyerbröker, 2021). However, numerous persistent barriers, many of which reflect broader challenges within the field, continue to limit its widespread adoption. In this section of the perspective paper, we summarize the most important critical issues that must be addressed to increase the uptake of VRET among therapists treating acrophobia. We also explore potential future developments that may overcome these barriers and consider possible solutions.

### 2.1 Technology level

Technological barriers to adapting VRET for treating acrophobia are quite general and similar to those in other psychological treatments. One of the primary technological challenges is the cost associated with the technology and its ability to deliver advanced, realistic experiences (Arnfred et al., 2023; Slater et al., 2020). Geographic location, ethnicity, gender, and socioeconomic status serve as barriers that perpetuate digital divides, excluding certain groups from access (Saeed and Masters, 2021). Technical infrastructure poses another barrier to VRET. In some regions, affordable or reliable broadband internet access is still unavailable, and even when it is, the average internet speed may limit the quality of streaming iVR content to unacceptable level (Ong et al., 2022). This issue affects not only individuals but also clinical practice institutions, as the high expenses involved in acquiring and maintaining iVR equipment make it challenging to implement this technology widely. While the price of iVR hardware has decreased substantially over the past few years, the expense of purchasing or licensing the software required to deliver VRET is a different matter. This issue is further compounded by the necessity of continuous software upgrades to ensure the application remains in optimal condition (Wray et al., 2023). Despite concerns about iVR’s costs among some mental health providers, evidence suggests that healthcare providers are generally willing to incur these expenses if they perceive a potential for improved patient outcomes. A systematic review supports that mental health providers are more accepting of telemedicine expenses when the technology enhances clinical effectiveness (Harst et al., 2019).

Another significant issue is the immaturity of interaction design techniques and user experience within iVR environments. Unlike non-immersive simulations, iVR requires a sophisticated synchronization of user movements, visuospatial cues, and both facial and bodily responses to create an effective therapeutic setting and understand reactions in relation to the phobic stimulus of a high-altitude environment. This complexity necessitates the integration of multiple technologies beyond standard iVR. All of these factors not only add to the complexity but also increase costs, posing financial challenges for the widespread adoption of VRET for acrophobia (Kampmann et al., 2016). The realistic representation and accuracy of these components are crucial for effective psychotherapy, as physical cues play a vital role in the interaction between therapists and clients during VRET (Arnfred et al., 2023; Ong et al., 2024). To address the immaturity of interaction design techniques and user experience within iVR environments, one potential solution is the development of advanced multimodal interaction systems that seamlessly integrate various sensory and motor inputs.

A critical aspect that is often underutilized in VRET is the integration of biofeedback mechanisms. While there are VRET studies where biosignals are acquired, they are typically monitored externally via a phone or another device, rather than being integrated into the iVR environment itself (Felnhofer et al., 2014; Moldoveanu et al., 2023). This lack of integration prevents both the client and therapist from sharing a fully immersive experience within the virtual phobic environment during VRET, thereby reducing the potential for a genuine sense of presence (Varšová et al., 2024). The obvious solution is the integration of biosignal monitoring directly within the iVR environment; however, we must also reconsider the associated costs. This could be achieved by embedding sensors into the iVR headset or by using wearable devices that track physiological responses such as heart rate (HR), skin conductance, and respiratory rate in real time, so that therapists and clients can adjust the course of therapy sessions based on this data.

### 2.2 Patient-therapist level

A crucial consideration in evaluating VRET is the role of the psychologist, whose presence may significantly influence therapy outcomes. While initial research focused on therapist-assisted iVR interventions for phobias (Kampmann et al., 2016; Anderson et al., 2013), recent advancements have explored the potential of automated therapy for acrophobia (Freeman et al., 2018; Bălan et al., 2021). However, the high dropout rates associated with self-led digital interventions (Krzystanek et al., 2021; Saad et al., 2021) highlight the importance of usability and therapist involvement. Evidence suggests that therapist-led VRET enhances treatment outcomes through the establishment of a therapeutic relationship and goal-setting (Buchholz and Abramowitz, 2020), which is also supported by recorded biosignal data (Varšová et al., 2024). Despite the promising potential of automated interventions, the value of human guidance remains a critical, yet under-researched, aspect of VRET for acrophobia.

Qualitative insights from Ong et al. (2024) reveal that therapists often find current VRET tools limited, particularly due to the lack of customizable iVR stimuli. For example, in treating acrophobia, some clients may struggle with open-height spaces rather than elevators, necessitating more tailored iVR experiences. Therapists emphasize the importance of incorporating client feedback and retaining these adjustments across sessions.

Another issue is that therapists’ attitudes toward VRET for acrophobia are mixed; while initial exposure may improve perceptions (Rimer et al., 2021), skepticism regarding its realism and effectiveness persists (Lindner, 2021). This skepticism, coupled with a preference for familiar therapeutic methods (Von Ranson et al., 2013), poses a barrier to wider adoption. Furthermore, concerns about iVR’s suitability for certain demographics, such as those prone to iVR-induced cybersickness (Kim et al., 2021; Pimentel et al., 2021), limit its application. However, many of these limitations have been mitigated in the commercially available head-mounted display systems currently on the market Research indicates that approximately 0.4% of users experience symptoms of simulator sickness with these modern systems, which represents a relatively low incidence (Kourtesis et al., 2019).

The collection and utilization of feedback from both clients and therapists are vital for refining VRET systems. However, many VRET applications lack thorough user testing, particularly with relevant participants—therapists and clients (Ong et al., 2024; Pedram et al., 2020). Feedback is essential for enhancing the virtual environment’s realism and interaction quality (Kouijzer et al., 2023; Levy et al., 2023).

## 3 Current state-of-the-art and possibilities for acrophobia via VRET

Given the current level of technology, VRET should evolve to offer more personalized and immersive experiences for clients with acrophobia. While advancements are already underway, critical gaps in research and practice remain. Addressing these gaps will transform VRET into a more powerful and effective tool for treating acrophobia.

### 3.1 Technology level

Current technological improvements can foster a more immersive and interactive environment, ultimately strengthening the therapeutic relationship in acrophobia treatment despite being mediated by iVR technology.

Continuous hardware and software innovations contribute to increasingly sophisticated iVR user experiences among multiple users. The Collaborative Immersive Virtual Environment (CIVE) platform, designed to facilitate interaction among multiple participants, offers potential benefits such as reduced waiting times and improved operational efficiency (Juřík et al., 2016; Šašinka et al., 2019; De Back et al., 2023; Petersen et al., 2023). CIVEs can be tailored to simulate real-life interactions, enhancing the dynamics of collaborative relationships (Zhang et al., 2020). However, there is a lack of comprehensive guidelines for designing virtual environments tailored to specific collaborative tasks for acrophobia (Wang et al., 2016). Regrettably, this particular issue has not garnered sufficient research attention.

The integration of biofeedback in iVR is a crucial area for enhancing VRET. Therapists highlight the importance of helping clients recognize their bodily responses as part of VRET for acrophobia. For instance, iVR environments could adapt in real-time to clients’ HR to facilitate relaxation training. Biofeedback also provides a clinically useful way to assess client affect, particularly since current iVR avatars may not yet accurately track facial expressions (yet). Using biosignal measurement tools such as HR and heart rate variability (HRV) calculation can deepen the understanding of anxiety-related experiences (Duong et al., 2020; Fiľo and Janoušek, 2022). These technologies allow therapists to identify significant in-session cues of acrophobic clients and optimize the therapeutic process, offering an objective assessment of the client’s mental state during VRET (Beutler and Harwood, 2004; Ihmig et al., 2020; Varšová et al., 2024).

Technical advancements in hardware, while challenging, are evolving rapidly. Integrating haptic interfaces and interactive elements, such as virtual objects or tools, can provide clients with a more engaging way to work through their phobias or anxieties (Arif et al., 2023; McAnally and Wallis, 2022). Recent developments in face and gaze tracking are particularly promising. HTC has released an iVR headset attachment to track facial expressions (Stein, 2021), and Facebook has published research on a prototype iVR headset that tracks eye movements (Matsuda et al., 2021). Upcoming versions of Oculus headsets also indicate the inclusion of face and eye-tracking features (Heaney, 2021). These advancements suggest that incorporating these features into VR-based therapies could be imminent and should be rapidly evaluated for clinical use in acrophobia. The question is, how soon can these advancements be integrated into psychotherapy practice and applications?

### 3.2 Patient-therapist context—collaboration along the way

The relationship between clients and therapists in VRET for acrophobia can be significantly enhanced through various innovative approaches. Beyond improving traditional therapeutic settings, there is substantial potential to advance remote therapy contexts as well. While face-to-face counseling is common for treating psychological conditions, including acrophobia, many patients face challenges accessing these services. Recent studies affirm that iVR interactions closely emulate in-person interactions (Rogers et al., 2022), presenting a promising alternative.

Currently, traditional telehealth methods, such as videoconferencing and telephone sessions, are widely used for remote psychotherapy. A study by Pedram et al. (2020) compared simulated counseling sessions via iVR and Skype, finding that iVR outperformed Skype in three crucial aspects: therapeutic efficacy, session realism, and sense of presence. These findings suggest that iVR can enhance the remote therapeutic experience more effectively than current telehealth methods. What remains is to apply this to VRET for acrophobia.

Building rapport and maintaining therapeutic relationships in iVR environments requires special attention, as discussed in previous sections on barriers (Glass and Bickler, 2021). Li and Yip’s (2023) case study, the first to combine CIVE and therapy, examined the feasibility of using a custom CIVE for remote arts therapy. Although this study provided preliminary insights into the advantages of iVR therapy conducted remotely, it did not specifically explore the potential of VRET and CIVE as an ideal meeting place for therapists and clients with specific fears, such as acrophobia.

No studies have yet investigated the connection between VRET and CIVE in this context, nor have they explored the design and use of an appropriate CIVE to enhance engagement for both clients and therapists. This gap represents a significant opportunity for future research. Both in-person and remote VRET sessions could become more immersive and engaging, increasing therapy participation when clients can collaborate with their therapists within a well-designed virtual therapeutic environment.

Integrating HRV measurement can greatly benefit both therapists and clients. However, without established guidelines to manage patients who become visibly upset in virtual scenarios, challenges arise. The lack of standardization in virtual environments and VRET makes it difficult for psychotherapists to effectively use iVR CBT and understand clients in phobic settings. While future research should address these issues, incorporating HRV monitoring is a promising step forward. It enables therapists to better understand clients’ physiological responses and respond appropriately—either by reducing the intensity of the environment or using calming techniques.

## 4 Discussion

The evolution of VRET for acrophobia holds significant promise but requires addressing current technological and practical barriers. Enhancing hardware and software capabilities, integrating real-time biofeedback, and developing CIVEs can make VRET more immersive and effective. Additionally, maintaining therapist involvement in VRET sessions is crucial for improving treatment outcomes. Future research should focus on creating and evaluating integrated VRET and CIVE systems to optimize patient-therapist interactions and therapeutic efficacy. Addressing these challenges will transform VRET into a more powerful and accessible tool for treating acrophobia.

## Data Availability

The original contributions presented in the study are included in the article/supplementary material, further inquiries can be directed to the corresponding author.
